# CHRONIC NEGATIVE SOCIAL TIES: IMPLICATIONS FOR EXPOSURE AND REACTIVITY TO DAILY SOCIAL INTERACTIONS

**DOI:** 10.1093/geroni/igac059.486

**Published:** 2022-12-20

**Authors:** Kira Birditt, Angela Turkelson, Richard Gonzalez, Toni Antonucci

**Affiliations:** University of Michigan, Ann Arbor, Michigan, United States; University of Michigan, Ann Arbor, Michigan, United States; University of Michigan, Ann Arbor, Michigan, United States; University of Michigan, Ann Arbor, Michigan, United States

## Abstract

Negative relationships (i.e., irritating, demanding) predict poor well-being and daily processes may account for these links. This study examined longitudinal trajectories of negative ties and their links with daily social interactions and well-being. A total of 169 individuals ages 33 to 91 reported negative relationship quality (spouse/child/friend) in 1992, 2005, 2015, and 2018 and completed 4-5 days of surveys every three hours regarding positive and negative social interactions and negative affect. Latent class growth models revealed two trajectories of negative relationships (moderate-stable and low-decreasing). Individuals in the moderate-stable trajectory reported more frequent daily interpersonal tensions but no link with positive interactions. The link between negative relationship trajectories and daily negative affect was moderated by daily positive interactions such that the association was reduced when individuals had positive interactions. Thus, negative ties may increase exposure but not reactivity to daily tensions and daily positive interactions buffer the negative tie-well-being link.

